# Adenomatoid Tumor of the Adrenal Gland: Report of Two Cases and Review of the Literature

**DOI:** 10.3389/fendo.2021.692553

**Published:** 2021-06-23

**Authors:** Jiexia Guan, Chang Zhao, Hengming Li, Wenjing Zhang, Weizhen Lin, Luying Tang, Jianning Chen

**Affiliations:** ^1^ Department of Pathology, The Third Affiliated Hospital of Sun Yat-Sen University, Guangzhou, China; ^2^ Department of Pathology, School of Basic Medical Sciences, Southern Medical University, Guangzhou, China

**Keywords:** adenomatoid tumor, adrenal gland, clinicopathological features, differential diagnosis, case report

## Abstract

Adenomatoid tumor (AT) is an uncommon benign neoplasm of mesothelial origin, usually occurring in the female and male genital tracts. Extragenital localization such as the adrenal gland is extremely rare. Until now, only 39 cases of adrenal AT have been reported in the English literature. Here we report two novel cases of adrenal AT that occurred in male patients aged 30 and 31 years. The tumors were discovered incidentally by computed tomography (CT). Macroscopically, the tumors were unilateral and solid, and the greatest dimension of the tumors was 3.5 and 8.0 cm, respectively. Histologically, the tumors consisted of angiomatoid, cystic, and solid patterns and infiltrated the adrenal cortical or medullary tissue. The tumor cells had low nuclear/cytoplasmic ratio, with no pathological mitosis or nuclear pleomorphism. Thread-like bridging strands and signet-ring-like cells could be seen. Immunohistochemically, the tumor cells were positive for epithelial markers (AE1/AE3, CK7) and mesothelial markers (D2-40, calretinin, and WT-1). The Ki-67 index was approximately 1 and 2%, respectively. The differential diagnosis of adrenal AT includes a variety of benign and malignant tumors. The patients had neither local recurrence nor distant metastasis at 21 and 8 months after removal of the tumor. In the literature review, we comprehensively summarized the clinical, morphological, immunohistochemical, and prognostic features of adrenal AT. Adrenal ATs are morphologically and immunophenotypically identical to those that occur in the genital tracts. Combining the histology with immunohistochemical profiles is very supportive in reaching the diagnosis of this benign tumor, helping to avoid misdiagnosis and overtreatment.

## Introduction

Adenomatoid tumor (AT) is a benign neoplasm originating from mesothelial cells. It is commonly encountered in the genital tracts, especially the uterus and fallopian tube in females and paratesticular sites in males (1, 2). Extragenital localization such as the adrenal gland is extremely rare. Until now, only 39 cases of adrenal AT have been reported in the English literature (3–32). In this paper, we report two novel cases and review the literature to summarize the clinical presentation, histological features, immunophenotype, differential diagnosis, and prognostic features of adrenal AT in order to comprehend this rare tumor better and avoid misdiagnosis.

## Materials and Methods

Case No. 1: A 30-year-old man admitted to the hospital complained of palpitation and dizziness for one year. The laboratory results, including routine blood examination, serum cortisol, and ketosteroid levels, were within the normal range. Computed tomography (CT) examination showed a relatively well-demarcated, oval mass measuring 34 × 24 mm in the right adrenal gland. A laparoscopic right adrenalectomy was performed. After surgery, the patient was discharged from the hospital and had an uneventful recovery. No recurrence or metastasis was observed during 21 months follow-up.

Case No. 2: A 31-year-old man presented to the hospital for a routine physical examination, and a mass lesion was incidentally detected by CT. He was asymptomatic, and CT demonstrated a left adrenal mass measuring 77 × 43 mm which was regarded as an adrenal adenoma. Preoperative laboratory examinations revealed that the urinary vanillylmandelic acid, serum cortisol, and ketosteroid levels were within normal limits. A laparoscopic left adrenalectomy was performed. The postsurgical recovery was also uneventful. The patient had no signs of recurrence or metastatic lesion after 8 months follow-up.

All the resected specimens were routinely fixed in 10% neutral buffered formalin and embedded in paraffin. Sections of 4 μm in thickness were stained with hematoxylin and eosin for histopathological examination. Immunohistochemical analyses were performed using the Ventana Ultra View Universal DAB Detection Kit, and primary antibodies were obtained from different companies (see **Table 1**). Clinical data were obtained, including patients’ gender, age, clinical symptom, tumor size, anatomic site of involvement, and follow-up. A review of the literature was also done *via* electronic searches through PubMed for articles that contained the following search terms, adenomatoid tumor and adrenal gland. Only those written in English were included.

**Table 1:** Summary of primary antibodies.

Antibody	Clone	Source	Dilution
AE1/AE3	AE1/AE3	Novocastra	1:100
CK7	OV-TL12/30	Maixin	1:100
Calretinin	5A5	Novocastra	1:100
D2-40	D2-40	Maixin	1:100
HBME-1	HBME-1	Maixin	1:50
WT-1	MX012	Maixin	1:50
HMB45	HMB45	Novocastra	1:100
Melan-A	A103	Novocastra	1:50
Desmin	DE-R-11	Novocastra	1:100
Actin	αsm-1	Novocastra	1:100
S100	4C4.9	Maixin	1:200
Ki-67	MIB-1	Maixin	1:150
Syn	27G12	Novocastra	1:200
CgA	MX018	Maixin	1:200
CD31	JC/70A	Maixin	1:100
CD34	QBEnd/10	Novocastra	1:200

## Results

### Pathological Findings

**Figure 1:**
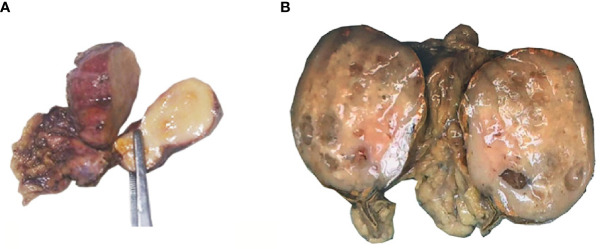
Gross examination of adrenal AT. **(A)** The tumor of case No. 1 measured 3.5 × 2.0 × 1.0 cm in size. Cut surface showed a solid, grayish yellow tumoral lesion. **(B)** The tumor of case No. 2 measured 8.0 × 6.5 × 3.0 cm in size. On cut surface, the tumor was solid, grayish yellow with some tiny thin-walled, translucent cysts. Small remnants of normal adrenal tissues were perceived at the periphery.

The excised adrenal tumor specimens measured 3.5 × 2.0 × 1.0 cm and 8.0 × 6.5 × 3.0 cm in size, respectively. The tumors were well circumscribed. On cut surface (**Figure 1**), the tumors were solid, grayish yellow without hemorrhage or necrosis. Some tiny thin-walled cysts were identified in the bigger mass. Small remnants of normal adrenal tissues were perceived at the periphery in two cases.

**Figure 2:**
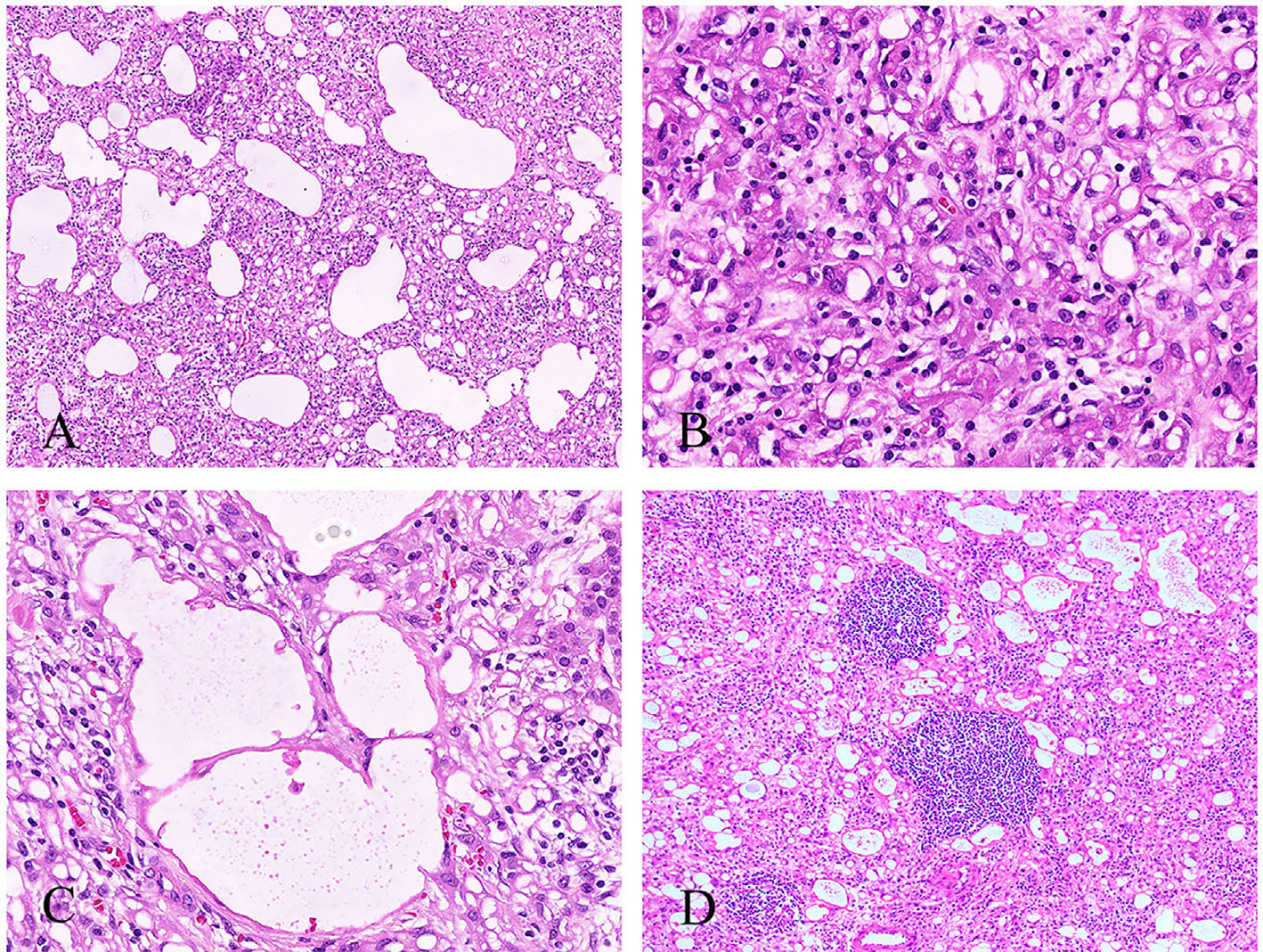
Histological features of the adrenal AT. **(A)** The angiomatoid pattern of tumor composed of anastomosing, variably sized tubules lined by flattened or cuboidal cells (100×). **(B)** The solid pattern of tumor composed of epithelioid cells with eosinophilic cytoplasm, and signet-ring-like cells can be seen (400×). **(C)** Thread-like bridging strands were found (400×). **(D)** Some lymphocytes were infiltrated and aggregated in the stroma (100×).

Histologically, the tumor cells infiltrated and compressed the normal adrenal cortical or medullary tissue, imparting an appearance of an infiltrative growth pattern. The invasion of the capsular or periadrenal adipose tissue was not detected. The tumors consisted of different patterns: angiomatoid, cystic, or solid. The angiomatoid pattern was composed of anastomosing glands or small to medium sized tubules lined by flattened or cuboidal cells with scant-to-moderate amounts of eosinophilic cytoplasm (**Figure 2A**). The cystic pattern was composed of large cysts lined by flattened or cuboidal cells. The solid area was covered with epithelial cells with abundant eosinophilic cytoplasm, and signet-ring-like cells could also be seen (**Figure 2B**). All the tumor cells had low nuclear/cytoplasmic ratio, with no appreciable mitotic activity or nuclear pleomorphism. Thread-like bridging strands were found in two cases (**Figure 2C**). No necrosis was observed. Lymphocytes infiltrated and aggregated in the stroma of two cases (**Figure 2D**).

**Figure 3:**
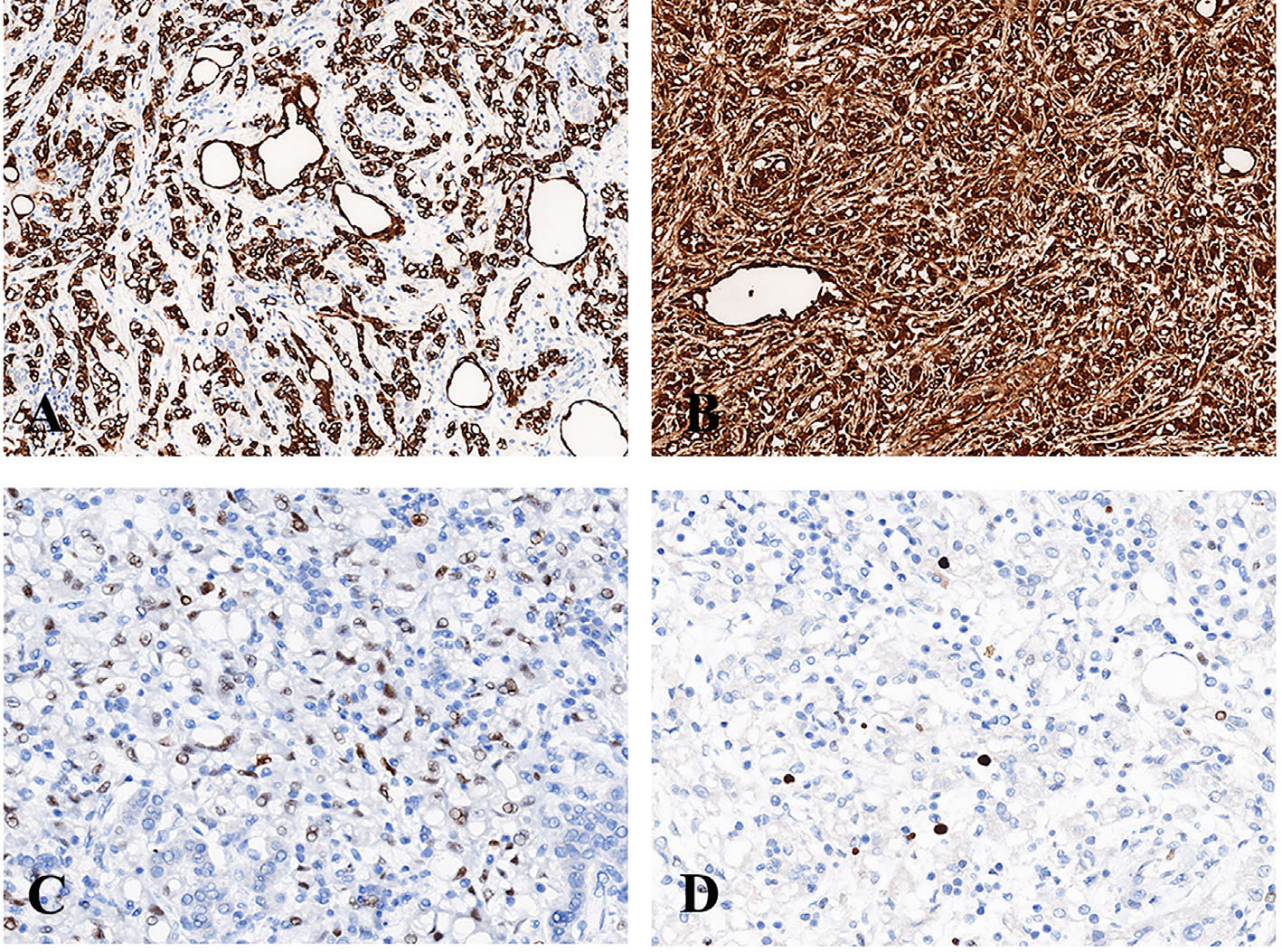
Immunohistochemistry of adrenal AT. **(A)** The tumor cells were diffusely positive for AE1/AE3 (200×). **(B)** The tumor cells were diffusely positive for calretinin (200×). **(C)** The tumor cells were focally positive for WT-1 (400×). **(D)** The Ki-67 index was approximately 2% (400×).

Immunohistochemically, the tumor cells of case No. 1 were positive for AE1/AE3, calretinin, D2-40, focal positive for HBME-1 and negative for HMB45, Melan-A, Desmin, Actin, and S100. The Ki-67 index was about 1%. Tumor cells of case No. 2 were positive for AE1/AE3 (**Figure 3A**), CK7, calretinin (**Figure 3B**), D2-40, focal positive for WT-1 (**Figure 3C**), and negative for HMB45, Melan-A, Desmin, S100, Syn, CgA, CD31, and CD34. The Ki-67 index was approximately 2% (**Figure 3D**).

In summary, two cases were diagnosed as adenomatoid tumor of the adrenal gland definitely when the clinical findings were combined with morphology and immunohistochemical results.

### Review of the Literature

By searching PubMed for adenomatoid tumor originating from the adrenal gland, we could find only 39 cases reported in the English medical literature to date. The clinicopathological features of this 39 previously reported adrenal ATs and our two cases are presented in **Table 2**.

**Table 2:** Clinicopathological features of adenomatoid tumors of the adrenal gland.

NO.	Author and citation	Age-gender-side	Greatest dimension (cm)	Clinical Findings and symptoms	Gross	Histologic patterns	Extension	Lymphocytes infiltrate/aggregate	Signet-ring-like cells	Positive IHC staining	Negative IHC staining	Follow-up
1	Evans et al. (3)	36-M-L	11.0	IRF (painless gross hematuria)	Solid-cystic	Papillary, glandular, cystic	No	Not stated	Yes	Not stated	Not stated	8 months
2	Travis et al. (4)	24-M-L	1.1	IRF (Cushing syndrome)	Solid-cystic	Cystic	Cortex and periadrenal adipose tissue	Not stated	Not stated	AE1/AE3, vimentin, EMA (weakly positive)	Not stated	6 months (died of pulmonary carcinoid)
3	Simpson (5)	44-M-L	3.2	IRF (hypertension)	Solid-cystic	Adenoid, angiomatoid, cystic, solid	Cortex, medulla and periadrenal adipose	Yes	Yes	AE1/AE3, CAM 5.2, CK7, vimentin, CK5/6 (weak and focal)	CD15, CD31, CD34, CK20, MOC31, CEA-P	177 months
4	Raaf et al. (6)	49-M-R	1.3	IFA	Solid	Classic	No	Not available	Not available	MAK-6, AE1/AE3, vimentin	Not stated	Found at autopsy
5	Raaf et al. (6)	57-M-L	3.8	IFA	Solid	Classic	No	Not available	Not available	MAK-6, AE1/AE3, vimentin	Not stated	Found at autopsy
6	Raaf et al. (6)	50-F-R	0.5	IFA	Solid	Classic	No	Not available	Not available	Not stated	Not stated	Found at autopsy
7	Raaf et al. (6)	40-M-L	6.0	IRF (CT scan during sarcoma staging)	Cystic	Cystic	No	Not available	Not available	MAK-6, AE1/AE3, vimentin	Not stated	Not stated
8	Angeles-Angeles et al. (7)	34-M-R	3.0	IFA (AIDS)	Solid	Round, oval, irregular, or tubular spaces, papillary	Cortex/medulla	Not stated	Not stated	Low molecular weight cytokeratin CKAE-3, vimentin (weak reaction)	CD34, FVIII	Found at autopsy (died of acute bilateral pneumonia)
9	Gasque et al. (8)	28-M-R	9.0	IRF (acute cholecystitis)	Solid-cystic	Adenoid, cystic	No	Yes	Not stated	CAM 5.2	Neuroendocrine markers	16 months
10	Glatz et al. (9)	54-M-L	6.5	IRF (pneumonia)	Solid-cystic	Papillae, tubular spaces and gland-like, solid	Cortex	Yes	Yes	Cam5.2, Lu-5, calretinin thrombomodulin(weakly)	CEA, MOC-31, BerEP4, CD34	Not stated
11	Isotalo et al. (10)	37-M-L	3.1	IFS (rectal adenocarcinoma)	Solid-cystic	Adenoid, angiomatoid, cystic, solid	Cortex, medulla and periadrenal adipose	Yes	Yes	AE1/AE3, CAM 5.2, CK7, vimentin, CK5/6 (weak and focal)	CD15, CD31, CD34, CK20, MOC31, CEA-P	40 months
12	Isotalo et al. (10)	31-M-R	3.2	IRF (asymptomatic)	Solid	Adenoid, angiomatoid, solid	Cortex/medulla	Yes	Yes	AE1/AE3, CAM 5.2, CK7, vimentin, CK5/6 (weak and focal)	CD15, CD31, CD34, CK20, MOC31, CEA-P	Not stated
13	Isotalo et al. (10)	31-M- Not stated	3.5	IRF (hypertension)	Solid	Adenoid, angiomatoid, solid	Cortex	Yes	Yes	AE1/AE3, CAM 5.2, CK7, vimentin, CK5/6 (weak and focal)	CD15, CD31, CD34, CK20, MOC31, CEA-P	50 months
14	Isotalo et al. (10)	64-M-L	1.2	IFA	Solid	Adenoid, angiomatoid, cystic	Cortex, medulla and periadrenal adipose	Yes	Yes	AE1/AE3, CAM 5.2, CK7, vimentin, CK5/6 (weak and focal)	CD15, CD31, CD34, CK20, MOC31, CEA-P	Found at autopsy
15	Chung-Park et al. (11)	51-M-R	3.0	IRF (hypertension of primary aldosteronism)	Solid	Anastomosing glands and cleft-like spaces, multiple microcysts and canalicular spaces	Cortex	Yes	Yes	Cytokeratin, calretinin	Syn, CgA, factor VIII, CD34, S-100	Not stated
16	Kim et al. (12)	33-M-L	1.7	IRF (hypertension)	Solid	Variably sized cystic spaces	Adrenal parenchyma	Not stated	Yes	Cytokeratin, calretinin	CD34	Not stated
17	Denicol et al. (13)	42-M-L	19.0	IRF (renal lithiasis, hypertension)	Solid-cystic	Small tubules, cysts or string-shaped	No	Not stated	Not stated	AE1/AE3, vimentin	CEA, CD31	3 years
18	Garg et al. (14)	46-M-L	Microscopical	IRF (central and right flank abdominal pain)	Cystic	Anastomosing glands and tubules	No	Yes	Yes	Calretinin, CK5/6	HMB45, myeloperoxidase	Not stated
19	Garg et al. (14)	33-M-L	1.7	IRF (hypertension)	Solid	Anastomosing glands and tubules	No	Yes	Yes	Calretinin, CK5/6	HMB45, CD34, myeloperoxidase	Not stated
20	Garg et al. (14)	33-M-R	4.2	IRF (asymptomatic)	Solid	Anastomosing glands and tubules	No	Yes	Yes	Calretinin	HMB45, CD34, myeloperoxidase	1 year
21	Varkarakis et al. (15)	54-M-R	3.6	IRF (renal lithiasis)	Solid	Sheets, tubules (with heterotopic ossification)	Cortex	Not stated	Not stated	Calretinin	Not stated	1 year
22	Hamamatsu et al. (16)	30-M-L	3.0	IFA	Solid	Anastomosing glands and various sized tubules	Cortex	Yes	No	Calretinin, D2-40, WT1, MC, CA125, vimentin, thrombomodulin, AE1/AE3, OV-TL 12/30, CAM5.2, MNF116	CD31, CD34, factor VIII-related antigen, CD56 ER, AR	Found at autopsy (acute coronary thrombosis)
23	Fan et al. (17)	42-M-L	2.5	IRF (hypertension, left renal cyst and nephrolithiasis)	Solid	Anastomosing, variably sized tubules, channels and small cystic	Cortex/medulla	Not stated	Yes	CK7, calretinin, EMA, antimesothelial cell antibody, vimentin	CEA, CD34, CD105, F8, VEGFR3	Not stated
24	Timonera et al. (18)	47-M-R	7.0	IRF (diverticulitis)	Solid	Anastomosing tubules and cystic spaces	Cortex	Yes	Not stated	D2-40, calretinin, CK5/6(weak reactivity)	Not stated	Not stated
25	Timonera et al. (18)	52-M-R	5.5	IRF (hypertension)	Solid-cystic	Anastomosing tubules and cystic spaces	Cortex	Yes	Not stated	D2-40 and calretinin, CK5/6(weak reactivity)	Not stated	Not stated
26	Hoffmann et al. (19)	26-M-R	15.0	IRF (asymptomatic)	Cystic	Glandular formations	No	Not stated	Not stated	Cytokeratin, calretinin	CD31, CD34, CD56	Not stated
27	Bisceglia et al. (20)	39-M-R	5.5	IRF (asymptomatic, cancer of the left colon 4 years ago)	Cystic	Variably sized, anastomosing tubules, channels, and small cystic spaces	Cortex/medulla	Yes	Yes	Pan keratins, CK5/6, calretinin	CD34, FVIII-RAg, CD31	Not stated
28	Chaudhry et al. (21)	60-M-R	11.0	IRF (hypertension, hyperlipidemia, and impaired fasting glucose)	Solid-cystic	Large cysts, fenestrated channels, and anastomosing tubules	No	Not stated	No	Calretinin, WT-1, pan-epithelial markers	Factor VIII, CD31, CD34	Not stated
29	Białas et al. (22)	29-M-R	4.0	IRF (asymptomatic)	Solid	Gland-like spaces and focal cystic dilation and solid nests	Cortex	Yes	Yes	AE1/AE3, CK7, calretinin, D2-40, vimentin, CK5/6(focal)	CD31, CD34, Factor VIII, CK20	Not stated
30	El-Daly et al. (23)	51-M-L	Not stated	IRF (asymptomatic)	Solid-cystic	Variably sized tubules and fenestrated channels	Cortex, capsule and periadrenal fat	Yes	Not stated	Calretinin, Cam5.2, CK7, vimentin, EMA (focally)	ER, CD31, CD34, Factor 8, CgA, Syn, S100, inhibin	Not stated
31	Liu et al. (24)	44-M-L	17.0	IRF (asymptomatic)	Cystic	Glandular and nest-like formations	No	Not stated	Not stated	Calretinin, EMA	CD34, CD56, HMB45	3 months
32	Phitayakorn et al. (25)	22-M-R	2.5	IRF (HIV infection)	Solid	Nests, cords, and tubules	enwrapped the ipsilateral renal artery and vein	Not stated	Yes	Calretinin, AE1/AE3, CAM 5.2	CD31, CD34, Factor VIII	7 months
33	Limbach et al. (26)	24-M-L	3.6	IRF (asymptomatic, SDHD mutation)	Solid	Anastomosing tubules and channels	Cortex/medulla, adrenal capsule and periadrenal fat	Yes	Yes	AE1/AE3, calretinin, WT-1, S100(focally)	Syn, CgA, CD31, CD34	6 months
34	Li et al. (27)	32-M-L	4.0	IRF (asymptomatic)	Solid	Cystic and sinusoid-like channels	Adjacent adrenal tissues	Yes	Yes	CK5/6, calretinin, D2-40, MC, vimentin, EMA (focally)	CgA, NSE, CD10	2.5 years
35	Zhao et al. (28)	62-M-R	3.0	IRF (hypertension)	Solid-cystic	Tubules, fenestrated channels, and small cystic spaces	Cortex, adrenal capsule and periadrenal adipose tissue	Yes	Not stated	AE1/AE3, CK5/6, calretinin,vimentin	CD31, CD34, FVIII, CEA, SMA, HMB45, Melan-A, S100	8 months
36	Sağlıcan et al. (29)	40-M-R	5.5	IRF (asymptomatic)	Solid-cystic	Papillary, microcysts and tubules	Cortex	Yes	Yes	AE1/AE3, calretinin	CD34, CD31	1 year
37	Krstevska et al. (30)	30-F-R	8.0	IRF (asymptomatic)	Cystic	Cystic spaces	No	Yes	Not stated	Vimentin, S100, MCA mesothelial Ag, CD 69	Actin, CK7, CD3	4 years
38	Jiang et al. (31)	26-M-R	4.0	IRF (asymptomatic)	Solid	Variably sized tubules and fenestrated channels	No	Not stated	Yes	CK5/6, calretinin, WT-1, D2-40, vimentin	CD34, CgA, Syn, S100	Not stated
39	Dietz et al. (32)	28-M-R	4.8	IRF (chronic abdominal pain)	Solid	Tubular, pseudoangiomatous	No	Yes	Yes	CK7, D2-40, BAP1, calretinin	Not stated	Not stated
40	our case	30-M-R	3.5	IRF (palpitation and dizziness)	Solid	angiomatoid, cystic and solid	Cortex/medulla	Yes	Yes	AE1/AE3, calretinin, D2-40, HBME-1 (focal)	HMB45, Melan-A, Desmin, Actin, S100	21 months
41	our case	31-M-L	8.0	IRF (asymptomatic)	Solid	angiomatoid, cystic and solid	Cortex/medulla	Yes	Yes	AE1/AE3, CK7, calretinin, D2-40, WT-1	HMB45, Melan-A, Desmin, S100, Syn, CgA, CD31, CD34	8 months

M, male; F, female, R, right adrenal gland; L, left adrenal gland; IRF, incidental radiographic finding; IFA, incidental finding during autopsy; IFS, incidental finding during surgery for unrelated reasons; Syn, synaptophysin; CgA, chromogranin, CK5/6, cytokeratin 5/6.

Patient’s age at diagnosis ranged from 22 to 64 years (mean 39 years, median 37 years), with more than half of the patients younger than 40 years of age. There are 18 cases of adrenal AT located in the left adrenal gland and 22 cases located in the right adrenal gland, and the location of one case was unavailable. ATs primarily occurred in males, accounting for 39 of 41cases.

All tumors presented in patients as incidental radiological, surgical, or autopsy findings. The tumor in thirty-four patients was discovered incidentally during radiological examinations, six patients during autopsy and one patient during surgery for resection of rectal adenocarcinoma. Most of the patients were asymptomatic and physical examination was non-specific. Some patients presented with symptoms or comorbidities. Ten cases of adrenal AT were found to be associated with hypertension. Three patients had nephrolithiasis. Two patients had a history of chronic abdominal pain. One patient suffered from painless gross hematuria. One patient of our case complained of palpitation and dizziness. The tumors in other patients were discovered during the investigation in the course of Cushing syndrome, sarcoma staging, acute cholecystitis, pneumonia, diverticulitis, inguinal hernia, and acquired immune deficiency syndrome (AIDS). Six cases of adrenal AT were found during autopsy, and the cause of death of four patients was not available. One patient with HIV-1 infection developed acute bilateral pneumonia with severe respiratory failure and died. The immediate cause of death was disseminated coccidioidomycosis. Another patient died of acute coronary thrombosis resulting in heart failure after drinking alcohol.

Macroscopically, the tumors ranged from 0.5 to 19.0 cm (mean 5.3 cm, median 3.8 cm) in greatest dimension. The dimension of two cases was unavailable. Twenty-three cases were presented as a solid mass, whereas twelve cases have been described as mixed solid and cystic, and only six cases as entirely cystic.

Histologically, extension of tumor into cortex/medulla or periadrenal adipose tissue was observed in 58.5% (24/41) of cases and one tumor enwrapped the ipsilateral renal artery and vein. Most of tumors contained multiple histologic patterns, such as adenoidal, angiomatoid, cystic, papillary, and solid patterns. Adenoidal, angiomatoid or cystic patterns were common in AT. Only 11 cases of tumor had solid patterns, and four cases had papillary patterns. All the tumor cells had low nuclear/cytoplasmic ratio, with no pathological mitosis or nuclear pleomorphism. Signet-ring-like cells were described in 23 cases, and lymphocytes infiltration or aggregates were observed in 25 cases. Ultrastructural study was done in 10 cases, and all displayed numerous long, bushy, and slender microvilli that were typical of mesothelial-derived cells.

Immunohistochemically, the tumor cells were positive for epithelial markers, such as AE1/AE3, CAM5.2, and CK7, and mesothelial markers, such as calretinin, D2-40, and WT-1. However, the tumor cells were weakly or focally positive for CK5/6, which was also the marker of mesothelial cells. Negative staining was observed for CD34, CD31, HMB45, Melan-A, Actin, Desmin, Syn, and CgA.

No local recurrence or metastasis of adrenal AT has ever been reported in 18 patients who were followed up for 3–177 months, and one patient died of pulmonary carcinoid.

## Discussion

Adenomatoid tumor (AT) of the adrenal gland is extremely rare. From 1988 to 2020, only 39 cases of adrenal AT were reported in the English medical literature, among which only four authors reported more than one case of adrenal AT (6, 10, 14, 18), and the rest was a single case report. Here we report two novel cases and review the literature to summarize the clinical, morphological, immunohistochemical, and prognostic features of adrenal AT more comprehensively than before.

Adrenal ATs tend to occur in adults in their fourth decade of life, but there is a wide age range. The tumor has a significant male predilection (male–female ratio, 39:2). A possible explanation related to the different roles of mesonephric ducts in males and females in embryological development together with the hypothesis that ATs arise from primitive mesenchymal cells associated with the Mullerian tract (33, 34). The mesonephric duct slowly transforms into a duct of epididymis and the paramesonephric ducts disappear in male, whereas the mesonephric ducts regress early during the embryogenesis in female (35). Therefore, it is conceivable that there is developing tissue that persists in males, so that this embryological difference between male and female systems may explain for the male predominance of adrenal AT. The ratio of adrenal AT located in the left and right adrenal gland was 18:22. It seems that the right adrenal gland was involved more frequently than the left adrenal gland which is contrary to the previous reports (11, 21). However, more cases are needed to draw a definite conclusion.

Usually, adrenal AT is non-functioning, asymptomatic and discovered incidentally during radiological examinations, surgical procedures for other unrelated reasons, or at autopsy. Radiological examinations are not specific for adrenal ATs. In rare occurrences, it is associated with symptoms or comorbidities, such as hypertension, AIDS, disseminated coccidioidomycosis, hematuria, Cushing syndrome, adrenal cysts, and kidney stones. About 24.4% (10/41) of adrenal AT was associated with hypertension. We speculate that adrenal AT may be associated with hypertension. However, the inherent relationship of hypertension and adrenal AT needs further investigation. Angeles-Angeles et al. (7) reported an autopsy case of an incidentally discovered AT arising in the right adrenal gland of a 34-year-old man with AIDS, and the immediate cause of death was disseminated coccidioidomycosis. Phitayakorn et al. (25) described another case of a 22-year-old man with AIDS who denied any symptoms and underwent a laparoscopic right adrenalectomy with no post-operative complications on follow-up examination seven months later. Patients with AIDS frequently present with a number of associated neoplasms, such as Kaposi’s sarcoma or lymphoma, often as a result of their immunosuppression. It is very unusual for patients with AIDS to present with adrenal gland neoplasms. One patient suffered from painless gross hematuria which was attributed to compression of the upper pole of the kidney by the adrenal AT (3). Fan et al. (17) reported a case of adrenal AT with a concurrent left kidney cyst and left nephrolithiasis in a man with moderate hypertension. Moreover, two cases of adrenal AT in a man who sought evaluation of renal colic or acute right flank pain due to nephrolithiasis were reported by Denicol et al. (13) and Varkarakis et al. (15), respectively. A few patients had a history of sarcoma (6), inguinal hernia (18), colon cancer (20), and mucoepidermoid carcinoma (11), and adrenal AT was incidentally discovered.

Macroscopically, the tumors are usually solid, but rarely, they present as solid-cystic or completely cystic tumors, which may probably be misdiagnosed as lymphangioma (10, 36). On the cut surface, the tumor is well circumscribed and grayish yellow. The average greatest dimension was 5.3 cm; only eight cases had reported with a greatest dimension of more than or equal to 8.0 cm, including one case reported by us.

Microscopically, the tumors may extend into the adrenal capsule, cortex, medulla, or extra-adrenal adipose tissue. We should note that this is not the diagnostic criterion of malignancy. The tumors have multiple patterns of growth, including adenoid, angiomatoid, cystic, solid, and papillary. About half of the cases showed two or more patterns (36). In our cases, the angiomatoid pattern is dominant, and solid or cystic areas can be found. Angiomatoid or cystic structures are often covered by flattened or cuboidal cells. In the solid structure, the tumor cells are epithelioid with eosinophilic cytoplasm, and signet-ring-like cells always can be seen. The papillary patterns are uncommon which are covered by flat or cuboidal cells (3, 7, 9, 29). There is no cellular atypia, pathological mitosis, or necrosis. An article proposed that thread-like bridging strands were a morphologic feature present in all adenomatoid tumors (37). Lymphocyte infiltration and aggregates can be found in most cases which is also the histological feature of adrenal ATs. Other histological characteristics have been noted including dystrophic calcifications (6, 10, 15–17, 19), intratumoral adipose tissue (14, 18, 22) and metaplastic ossification (15). Timonera et al. (18) reported two cases of composite AT and myelolipoma with unclear pathogenesis. In the non-neoplastic adrenal tissue, adrenocortical hyperplasia was found in two cases (4, 11), and cytomegalovirus (CMV) infections with classical viral inclusions were seen in a patient with AIDS (7).

Immunostaining of tumor cells is usually diffusely positive for epithelial markers (AE1/AE3, CAM5.2, CK7) (10, 36, 38) and is also positive for mesothelial markers, such as D2-40, calretinin, WT-1, and HBME-1. D2-40 and calretinin had been shown to be expressed in 100% of the cases (38–40), and the positive rate of WT-1 reached 95.5% (38). Therefore, calretinin, D2-40 and WT-1 are relatively specific markers of ATs. There is little research on molecular genetics of adrenal ATs. Only one reported adrenal AT case was found in a patient with a personal and family history of succinate dehydrogenase complex subunit D (SDHD) mutation (26). Further studies are required to explore the molecular genetics of ATs.

Because of the unusual location, lack of specific clinical and radiological features, the blurred boundary between the tumor and the adjacent tissue, existence of various growth patterns, adrenal ATs may lead to difficulties in diagnosis and need to be differentiated from other tumors. Differential diagnosis covers a variety of entities of benign and malignant tumors.

Benign tumors mainly include lymphangioma and angiomyolipoma. Lymphangioma is a benign hamartomatous tumor which is characterized by abnormal proliferation of lymphatic vessels that usually present as cystic masses, with hemangioma, cystic architecture, and lymphocyte aggregates similar to ATs, but lymphangioma shows absence of a solid pattern and is negative for epithelial markers, WT-1 and calretinin (41). Angiomyolipoma is a benign mesenchymal neoplasm composed of an admixture of thick dysmorphic blood vessels, smooth muscle, and adipose tissue. It is similar to the angiomatoid pattern of AT if the main component of angiomyolipoma is adipose tissue. However, tumor cells of angiomyolipoma are positive for HMB45, Melan-A, Actin, Desmin, and S100 (42).

The malignant tumors include epithelioid hemangioendothelioma, malignant mesothelioma (MM), and metastatic adenocarcinoma. Epithelioid hemangioendothelioma is a vascular neoplasm with cords or small nests of round endothelial cells with abundant eosinophilic cytoplasm (43). Tumor cells often have intracytoplasmic vacuoles representing small vascular lumina, like the signet-ring-like cells. Immunohistochemistry will be indicative of vascular origin which is positive for ERG, CD31, CD34, and Fli-1. MM is a malignant neoplasm of mesothelial origin. The immunohistochemistry of MM is the same as that of ATs. However, MM shows more atypia, easily visible mitosis, and higher proliferation index. P16/CDKN2A deletions are common genetic alterations in MM (44). Finally, the glandular-like pattern and signet-ring-like cells of ATs possibly raise suspicion of metastatic adenocarcinoma. However, the absence of significant atypia and the mesothelial immunoprofile of ATs help in avoiding misdiagnosis.

As for treatment, surgery is the main treatment, and postoperative adjuvant treatment is not required. Although only limited clinical follow-up data is available, no local recurrence or metastasis has been reported in adrenal ATs. In our two cases, patients have a good prognosis after surgery.

## Conclusion

Adrenal AT is a benign neoplasm of mesothelial derivation. The tumors tend to occur in adults in their fourth decade of life and have a significant male predilection. Most of the tumors are asymptomatic, discovered incidentally by imaging during surgery or at autopsy. The tumors are well circumscribed and solid, with a grayish yellow cut surface. Histologically, the tumor may extend into the adrenal capsule, cortex, medulla, or extra-adrenal adipose tissue. There is a mixture of multiple patterns of growth, including adenoid, angiomatoid, cystic, solid, and papillary. No cellular atypia, pathological mitosis, or necrosis can be seen. Signet-ring-like cells and thread-like bridging strands can be found. Lymphocyte infiltration and aggregates also exist. The tumor cells are positive for epithelial markers (AE1/AE3, CK7, CAM5.2) and mesothelial markers (D2-40, calretinin and WT-1). The differential diagnosis includes lymphangioma, angiomyolipoma, epithelioid hemangioendothelioma, malignant mesothelioma, and metastatic adenocarcinoma. Complete excision is the main treatment. No recurrence or metastasis has been reported. Histology and immunohistochemical profiles are very supportive in reaching the diagnosis of adrenal ATs, helping to avoid misdiagnosis and overtreatment.

## Data Availability Statement

The original contributions presented in the study are included in the article/supplementary material. Further inquiries can be directed to the corresponding author.

## Ethics Statement

Ethical review and approval was not required for the study on human participants in accordance with the local legislation and institutional requirements. The patients/participants provided their written informed consent to participate in this study. Written informed consent was obtained from the individual(s) for the publication of any potentially identifiable images or data included in this article.

## Author Contributions

JG and CZ: conceived the idea of the work, contributed significantly to analysis and manuscript preparation, performed the data analyses, and wrote the manuscript. HL: contributed to the manuscript preparation and reference collection, performed the data analyses. WZ and WL: stained sections of 4 μm in thickness with hematoxylin and eosin, performed the experiment of immunohistochemical analyses. LT: provided revision opinions and revised the manuscript. JC: supervised the study, funding support, and revised the manuscript. All authors contributed to the article and approved the submitted version.

## Funding

This work was supported by the Natural Science Foundation of Guangdong Province (2018A030313650), the Guangzhou Science and Technology Project (202102010156) and the NSFC cultivating grant of The Third Affiliated Hospital, Sun Yat-sen University (2020GZRPYMS01).

## Conflict of Interest

The authors declare that the research was conducted in the absence of any commercial or financial relationships that could be construed as a potential conflict of interest.
